# Altered Spontaneous Brain Activity in Patients With Diabetic Osteoporosis Using Regional Homogeneity: A Resting-State Functional Magnetic Resonance Imaging Study

**DOI:** 10.3389/fnagi.2022.851929

**Published:** 2022-05-06

**Authors:** Min Liu, Jiang Li, Juan Li, Hui Yang, Qianqian Yao, Xiuzhu Zheng, Zheng Zhang, Jian Qin

**Affiliations:** Department of Radiology, The Second Affiliated Hospital of Shandong First Medical University, Taian, China

**Keywords:** diabetic osteoporosis (DOP), type 2 diabetes mellitus, regional homogeneity, resting-state functional magnetic resonance imaging, cognitive impairment

## Abstract

**Background:**

The pathophysiological mechanism of cognitive impairment by osteoporosis in type 2 diabetes mellitus (T2DM) remains unclear. This study aims to further investigate the regional spontaneous brain activity changes of patients with diabetic osteoporosis (DOP), and the correlation between abnormal brain regions and bone metabolites.

**Methods:**

A total of 29 subjects with T2DM were recruited, including fourteen patients with DOP and thirteen patients without osteoporosis (Control group). Based on the resting-state functional magnetic resonance imaging (rs-fMRI) datasets acquired from all the subjects, a two-sample *t*-test was performed on individual normalized regional homogeneity (ReHo) maps. Spearman correlation analysis was performed between the abnormal ReHo regions with the clinical parameters and Montreal Cognitive Assessment (MOCA) scores.

**Results:**

In the DOP group, we demonstrated the significantly increased ReHo values in the left middle temporal gyrus (MTG), right superior occipital gyrus (SOG), aright superior parietal lobule (SPL), right angular gyrus (AG), and left precuneus (PE). Additionally, we also found a significant positive correlation between increased ReHo values in the left MTG and the average bone mineral density (BMD AVG), and average T scores (T AVG). The ReHo values of the right SOG and right SPL showed a negative correlation with MOCA scores, as well as a negative correlation between increased ReHo values in the right SPL and osteocalcin (OC) level.

**Conclusion:**

Patients with DOP showed increased spontaneous activity in multiple brain regions. The results indicated that osteoporosis exacerbated cognitive impairment and brain damage. Also, the OC might be considered as a bone marker to track the progression of cognitive impairment.

## Introduction

The International Diabetes Federation has released new estimates on the prevalence of diabetes worldwide, indicating that the number of diabetes patients was about 415 million and predicted that it would reach up to 642 million by 2040 (Ogurtsova et al., 2017). Diabetes can cause a variety of complications, such as microvascular disease, retinopathy, kidney disease, peripheral neuropathy, osteoporosis, and cognitive impairment (Tiehuis et al., 2010; Macpherson et al., 2017; Cho et al., 2018). Especially, diabetic osteoporosis (DOP) is a serious complications of diabetes mellitus, which is characterized by a reduction of bone mineral density (BMD), destruction of bone microstructural, and a high risk of fractures (Saito and Marumo, 2010; Hamann et al., 2012). Kostev et al. (2018) showed that osteoporosis was a risk factor for dementia. Lee et al. (2012) found that reduced BMD was associated with cognitive impairment and proposed that there was a correlation between cognitive status and BMD in postmenopausal women. However, it is unknown whether osteoporosis will exacerbate cognitive impairment and brain damage in patients with diabetes. The pathophysiological mechanism of cognitive impairment by osteoporosis in diabetes mellitus remains unclear. And whether some markers of bone metabolism may be key factors affecting cognitive ability.

A variety of bone-related peptides are secreted into the circulation, and whether they affect the central nervous system has attracted the attention of researchers (Chamouni et al., 2015). Revealing the spontaneous brain activity induced by osteoporosis helps to elucidate the neuropathological mechanism of cognitive dysfunction. In the present research, we compared the altered spontaneous brain activity between osteoporosis and non-osteoporosis patients with type 2 diabetes mellitus (T2DM) by regional homogeneity (ReHo) of resting-state functional magnetic resonance imaging (rs-fMRI), to explore the correlation of altered ReHo values with BMD, OC, and the neurocognitive scale, and to discuss the influence mechanism of osteoporosis. The findings may provide insight into the neurological underpinnings of osteoporosis-related brain dysfunction.

## Methods

### Subjects

The present study was approved by the ethics review committee of the second affiliated hospital of Shandong's first medical university. Informed written consent was obtained from each participant. The subjects who met the inclusion criteria in this study were recruited from June 2020 to September 2021 in the surrounding community by posting a recruitment notice. Twenty-nine right-handed Patients with T2DM participated in this study. Fifteen patients (5 male and 10 female, mean age 58.33 ± 4.63) were included in the DOP group, and fourteen non-osteoporosis patients (6 male and 8 female, mean age 56.00 ± 4.22) with matching gender, age, duration, and education were enrolled as the Control group. All patients maintained stable blood glucose by using oral drugs or insulin. The diagnosis of T2DM was based on standard criteria from the American Diabetes Association (2018). The criteria for the diagnosis of diabetes: The fasting plasma glucose (FPG) ≥126 mg/dL (7.0 mmol/L), fasting is defined as no caloric intake for at least 8 h or 2-h Plasma Glucose (PG) ≥ 200 mg/dL (11.1 mmol/L) during a 75-g oral glucose tolerance test (OGTT), the test should be performed as described by the WHO, using a glucose load containing the equivalent of 75-g anhydrous glucose dissolved in water or A1C ≥ 6.5% (48 mmol/mol), the test should be performed in a laboratory using a method that is NGSP certified and standardized to the DCCT assay or In a patient with classic symptoms of hyperglycemia or hyperglycemic crisis, random plasma glucose ≥200 mg/dL(11.1 mmol/L). The exclusion criteria included the following: (1) Patients with other complications of diabetes, such as severe liver and kidney dysfunction, diabetic retinopathy, diabetic peripheral neuropathy, etc; (2) diseases affecting bone metabolisms, such as hyperthyroidism, hypercortisolism, connective tissue disease, and glucocorticoid administration; (3) lesions in the brain, such as tumors, cerebral infarction, hemorrhage, or vascular malformation; (4) contraindication to MRI examination, such as the presence of metallic implants or claustrophobia; (5) a history of neurological or psychiatric disorders; (6) used bisphosphonates, calcium, vitamin D and other osteoporosis drugs in the last 3 months; (7) used hormone drugs; (8) other types of diabetes. People with one of the above conditions will be excluded from this experiment.

### General Information and Cognitive Assessment

Clinical examinations including measurements of height, weight, body mass index (BMI), glycosylated hemoglobin A1C (HbA1c), duration, and serum OC were carefully performed by specialists. All subjects underwent the Montreal Cognitive Assessment (MoCA). The MoCA is commonly used to screen for Mild Cognitive Impairment (MCI), for which it displays high-sensitivity (Hobson, 2015), with a final score ≥ 26 considered normal. Subjects with less than 12 years of education receive an extra point. MoCA testing took place in a quiet room by trained professionals.

### Bone Mineral Density (BMD)

The BMD was measured by dual-energy X-ray absorptiometry (Horizon W, Hologic Inc, US). The criteria for diagnosing osteoporosis using DXA are the following: scans of the lumbar spine and hip, selecting the L1 to L4 vertebral bodies and the femoral neck and total hip as a region of interest (ROI), and using the lowest T-score amongst the 3 ROIs to make the diagnosis (Cheng et al., 2020). According to diagnostic criteria of osteoporosis (Camacho et al., 2020): a T-score ≥ −1 indicated normal; T-score between −1 and −2.5 indicated osteopenia; and T-score was −2.5 or below indicated osteoporosis. The patients with DOP included met the diagnostic criteria for osteoporosis.

### MRI Data Acquisition

Images were acquired on a 3.0T MRI scanner (Discovery MR750, GE Healthcare, Waukesha, WI, USA) using a commercial eight-channel head coil. Foam padding was used to restrict head movement and earplugs were used to minimize scanner noise. Subjects were asked to lie with their eyes closed, not to fall asleep, and not to think of anything in particular. Routine sequence scanning was performed to exclude brain tumors, cerebral infarction, cerebral hemorrhage, and other brain abnormalities. High-resolution anatomical images were obtained with a sagittal T1-3D brain volume imaging sequence (TE = 3.2 ms, TR = 8.2 ms, TI = 450 ms, FOV = 256 × 256 mm, matrix = 256 × 256, layer spacing = 0 mm, layer thickness = 1 mm, layer number = 176, NEX = 1). Functional images were obtained axially using a gradient-echo planar imaging sequence with the following parameters: TR = 2,000 ms, TE = 30 ms, Slice = 41, Slice thickness = 3 mm, FA = 90°, matrix = 64 × 64, FOV = 224 × 224 mm, NEX = 1, time point is 240, number of slices: 41, scanning time = 8 min.

### Data Preprocessing and ReHo Analysis

Functional image preprocessing was performed using the data processing assistant in the rs-fMRI toolbox (Yan et al., 2016) (DPABI, http://rfmri.org/dpabi V6.0_210501). The first 10 time points were discarded, and the subjects with more than 2 mm maximum displacement in any dimension and 2 degrees of angular motion during the entire fMRI were excluded (one subject from each group was eliminated in this step). The functional images were then spatially normalized to standard coordinates and resampled to 3 × 3 × 3 mm^3^. After that, the linear trend of the time series was removed and a temporal filter (0.01 Hz < f < 0.08 Hz) was conducted to reduce the effects of low-frequency drift and physiological high-frequency noise.

Individual ReHo maps were generated by calculating Kendall's coefficient of concordance (KCC) of the time series of a given voxel to its nearest 26 voxels (Zang et al., 2004). The average ReHo values of all voxels in the significant region were extracted using the rs-fMRI data analysis tool (Song et al., 2011) (REST, http://www.restfmri.net/forum/REST_V1.8) in the mask generated by the standardized step. Then, the resulting data were spatially smoothed with a Gaussian kernel (fullwidth at half-maximum, FWHM = 6 mm). Finally, a z-transformation was conducted on the individual ReHo maps to generate normally distributed szReHo maps.

### Statistical Analysis

SPSS Statistics version 23.0 was used for statistical analysis. The inter-group comparison of nominal variables (sex) was performed using the *Chi-square* (χ^2^) test. Then, the *Kolmogorov-Smirnov* test was applied in each group to verify the normality of the other numerical data distribution. According to the normality or non-normality, the two-sample *t-*test and the *Mann–Whitney U* test were applied to reveal significant differences between the DOP group and the control group. To explore the inter-group ReHo differences, a two-sample *t-*test was performed on the individual normalized ReHo maps. And We used the false discovery rate (*FDR, p*<*0.01*) to correct the multiple comparisons for the *p-value*. *Spearman* correlation analysis was performed between the abnormal ReHo regions with the clinical parameters, such as BMD AVG, T AVG, MOCA, OC, and HbA1c, *p* < 0.05 was considered statistically significant.

## Results

### Demographics

According to the two-sample *t-*test, significantly decreased levels of BMD AVG, T AVG, MOCA scores, and OC were observed in the DOP group compared to the Control group (*p* < 0.05). There were no significant differences between the DOP group and the Control group in terms of age, sex, height, weight, BMI, HbA1c, duration, and education years (*p* > 0.05, Table 1).

**Table 1:** Demographic, clinical, and cognitive data.

	**DOP group** **(*n* = 14)**	**Control group** **(*n* = 13)**	***P* value**
Age (years)	58.33 ± 4.63	56.00 ± 4.22	0.169
Sex (male/female)	5/10	6/8	0.597^a^
HbA1c (%)	8.24 ± 1.21	8.64 ± 1.63	0.463
duration (month)	49.60 ± 6.51	50.00 ± 6.56	0.870
Education (years)	9.93 ± 2.40	11.86 ± 3.21	0.077
BMI (kg/m^2^)	23.90 ± 3.42	26.21 ± 3.34	0.077
Weight (Kg)	62.46 ± 9.83	68.32 ± 5.35	0.059
Height (CM)	161.60 ± 6.10	163.43 ± 5.14	0.392
BMD AVG (g/cm^2^)	0.77 (0.70,0.83)	1.04 (1.02,1.12)	0.000^*^^b^
T AVG	−2.80 (−3.30,−2.60)	−0.05 (−0.50,0.35)	0.000^*^^b^
MOCA	21.67 ± 2.47	25.64 ± 2.65	0.000^*^
OC (ng/ml)	23.53 (22.05,24.81)	29.94 (26.97,32.79)	0.000^*^^b^

* *p < 0.05. Data are presented as n for proportions, means ± SD for normally distributed continuous data, and median (QR) for non-normally distributed data*;

a *The p-value for sex was obtained using the χ2 test*;

b *The p-value was obtained using the Mann–Whitney U test; DOP, T2DM with osteoporosis;T2DM with non-osteoporosis as controls; BMI, body mass index; BMD, Bone Mineral Density; MOCA, Montreal Cognitive Assessment; OC, osteocalcin*.

### ReHo Differences and Correlation Analysis

**Figure 1:**
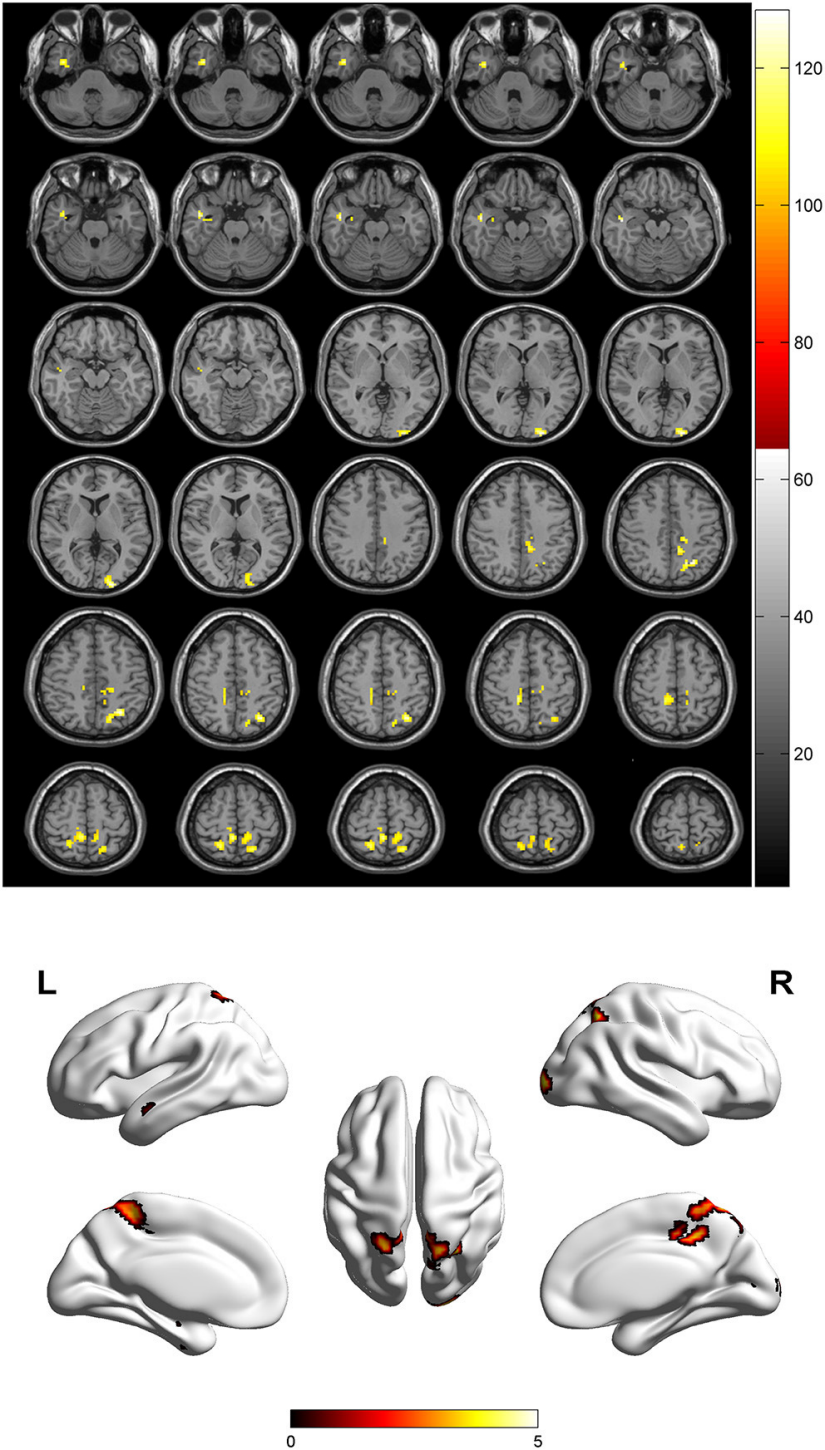
Spontaneous brain activity in patients with DOP group vs. Control group. The threshold was set *p* < 0.01(FDR correction).

**Figure 2:**
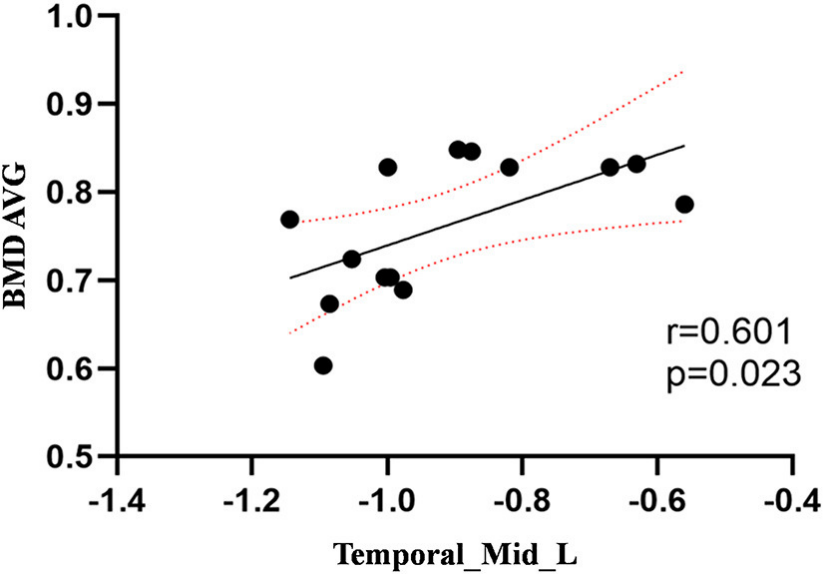
Correlation between ReHo values of the left MTG and the BMD AVG. The ReHo values of the left MTG showed a positive correlation with the BMD AVG (*r* = 0.601; *P* = 0.023).

**Figure 3:**
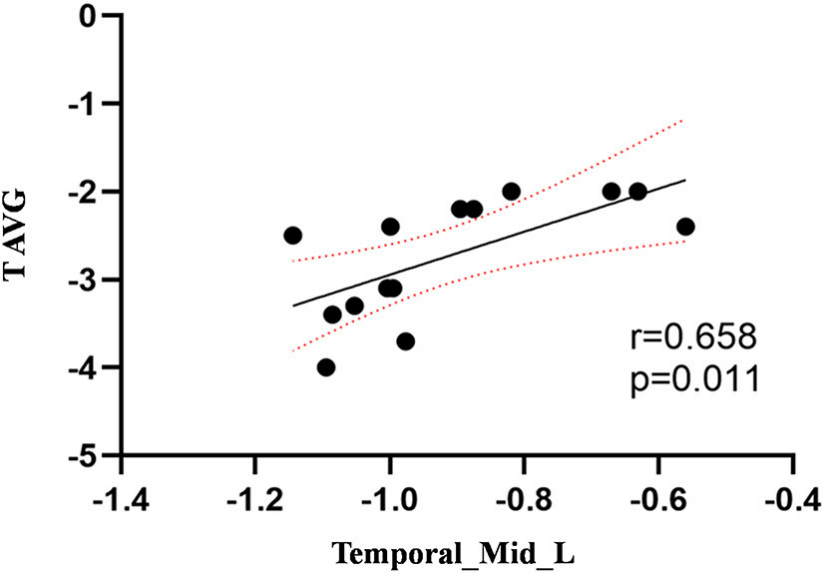
Correlation between ReHo values of the left MTG and the T AVG. The ReHo values of the left MTG showed a positive correlation with the T AVG (*r* = 0.658; *P* = 0.011).

**Figure 4:**
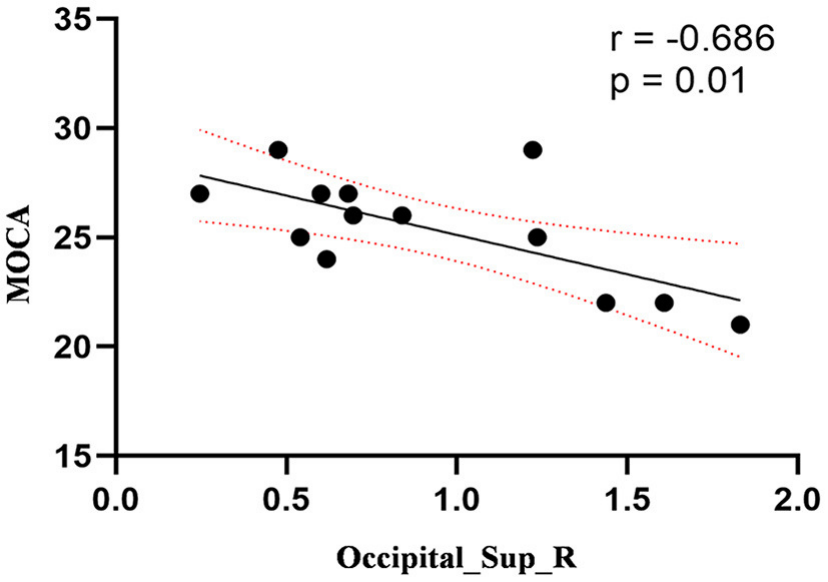
Correlation between ReHo values of the right SOG and the MOCA scores. The ReHo values of right SOG showed a negnative correlation with MOCA scores (*r* = −0.686; *P* = 0.01).

**Figure 5:**
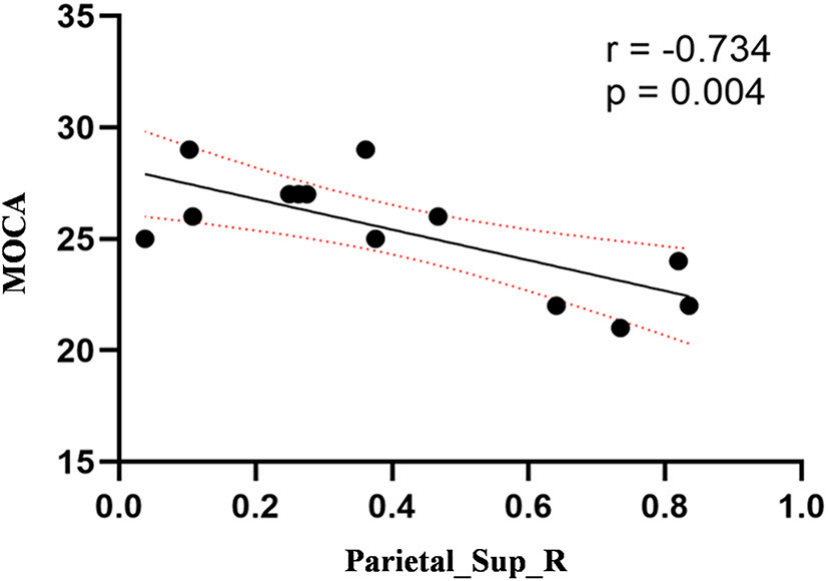
Correlation between ReHo values of the right SPL and the MOCA scores. The ReHo values of right SPL showed a negnative correlation with MOCA scores (*r* = −0.734; *P* = 0.004).

**Figure 6:**
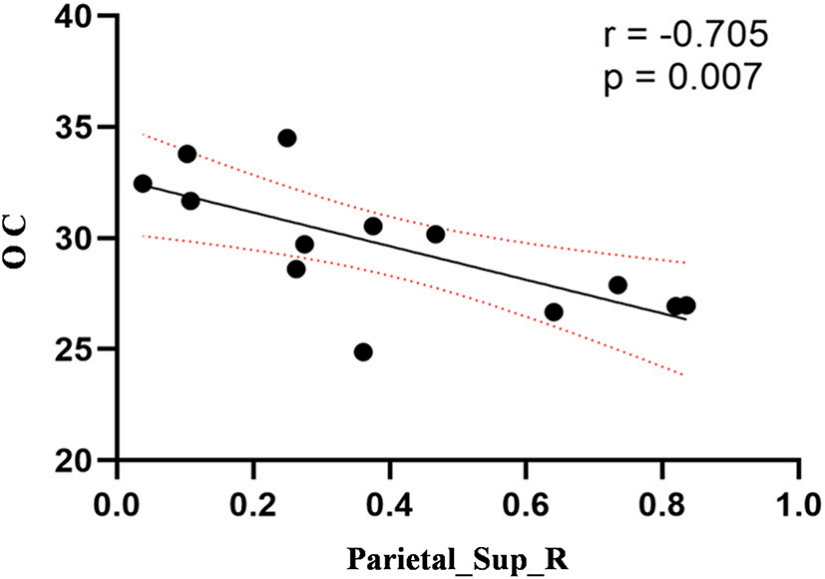
Correlation between ReHo values of the right SPL and OC. The ReHo values of right SPL showed a negnative correlation with OC (*r* = −0.705; *P* = 0.007).

Compared to the control group, patients with DOP had significantly higher ReHo values in the left middle temporal gyrus (MTG), right superior occipital gyrus (SOG), right superior parietal lobule (SPL), right angular gyrus (AG) and left precuneus (PE),as shown in Figure 1, Table 2. In the DOP group, the ReHo values of the left MTG showed positive correlation with the average BMD (BMD AVG) and average T scores (T AVG) (separately, *r* = 0.601, *P* = 0.023; *r* = 0.658, *P* = 0.011) (Figures 2, 3). The ReHo values of the right SOG and right SPL showed negative correlation with MOCA scores (separately, *r* = −0.686, *P* = 0.01; *r* = −0.734, *P* = 0.004) (Figures 4, 5). The ReHo values of right SPL showed negative correlation with OC (*r* = −0.705, *P* = 0.007) (Figure 6).

**Table 2:** Brain areas with significantly different regional homogeneity (ReHo) values between groups.

**Brain region**	**MNI coordinates**			**T value**	**Cluster Size**
	**X**	**Y**	**Z**		
Temporal_Mid_L (aal)	−48	−6	−21	4.4099	56
Occipital_Sup_R (aal)	24	−99	3	4.3332	62
Parietal_Sup_R (aal)	18	−60	63	3.4653	113
Angular_R (aal)	27	−60	48	4.3883	60
Precuneus_L (aal)	−15	−39	54	3.7383	118

*L, left; R, right; AAL, Anatomical Automatic Labeling; MNI, Montreal Neurological Institute; cluster_size > 50*.

## Discussion

In the present study, we demonstrated that patients with DOP showed significantly higher ReHo values in the left MTG, right SOG, right SPL, right AG, and left PE. In the DOP group, the ReHo values of the left MTG showed a positive correlation with the BMD AVG and T AVG, and the ReHo values of the right SOG and right SPL showed a negative correlation with MOCA scores, the ReHo values of right SPL showed negative correlation with OC level.

The temporal lobe is involved in audiovisual language integration and is considered a key component of the midline cortex (Ye et al., 2017). Impairment of the temporal lobe is considered to cause a defect in self-awareness (Chavoix and Insausti, 2017). Deletion of temporal lobe neurons and abnormal connections in the frontotemporal gyrus is thought to be closely related to dementia (Sato and Morishita, 2014). This study demonstrated that the ReHo values in the left MTG were increased in the DOP group, suggesting a decrease in the presence of cognitive function in patients with DOP. Furthermore, Zhou et al. (2014b) revealed that the regional function of the inferior and middle temporal gyrus was increased in mild cognitive impairment in patients with T2DM, which was linked to the functional compensation for cognitive decline. Therefore, we considered that osteoporosis exacerbated cognitive impairment, and the increased ReHo values in the left MTG were compensation mechanisms for cognitive impairment. In this study, we also discovered that the ReHo values of the left MTG showed a positive correlation with the BMD AVG and T AVG and confirmed that osteoporosis aggravated cognitive impairment based on T2DM. Lee et al. (2012) confirmed that cognitive impairment was associated with lower BMD, which was consistent with our findings.

The occipital cortex is usually considered to be the visual area, especially the primary visual cortex (Wandell, 1999), responsible for visual memory and vision processing (Rehman and Al Khalili, 2021). The occipital visual areas and frontal/parietal sensorimotor areas are activated concurrently during the visuospatial working memory task (Kwon et al., 2002). Functional impairment of the occipital lobe can lead to defects in the visual pathway, which affects the processing of visual information and recognition of the outside world (Tohid et al., 2015). This study demonstrated that the ReHo value in the right SOG was increased in the DOP group, and we held that the increased ReHo in the occipital cortex might be an important factor and early indicator of cognitive deficits and visual impairment in patients with DOP. Moreover, multiple studies have confirmed that the visual processing area of the occipital lobe was the most vulnerable region of the brain to T2DM (Cui et al., 2014; Wang et al., 2017). Therefore, we speculated that osteoporosis exacerbated visual impairment based on T2DM. We also found the ReHo values of the right SOG showed a negative correlation with MOCA scores, which represented an early compensatory mechanism for neuroplasticity induced by osteoporosis to counteract the effects of cognitive deficits and maintain normal cognitive functions probably, namely the greater the cognitive decline, the stronger the compensation (Wang et al., 2014).

In addition, we discovered that abnormities also occurred in the right AG and right SPL of the DOP group. As the important regions of the middle longitudinal fascicle (M dLF), AG and SPL encompassed numerous and specialized subdivisions that subserved a vast array of functions, which involved linguistic, attentive, visuospatial, and integrative audiovisual parts (Makris et al., 2013). According to a study of causal connectivity regarding subjective cognitive decline (SCD) (Cai et al., 2020), the right AG and SPL both had exhibited significant aberrant connections, which might reflect the impairments in cognitive functions. Tan et al. (2019) found the aberrant functional connectivity of the posterior cingulate cortex (PCC) to AG (associated with short-term memory functional decline) and SPL (associated with attention and spatial orientation) in patients with T2DM, thus causing the cognitive dysfunction. The AG is involved in a variety of cognitive processes, especially episodic memory retrieval and semantic processing, which has been found in human beings (Seghier, 2013; Ramanan et al., 2018; Qi et al., 2021). Therefore, we held that an increased ReHo in the right AG might be the important factors and indicator of episodic memory impairment and semantic processing disorder in the DOP group. Furthermore, we also discovered abnormities that occurred in the right SPL of the DOP group. It has been well established that SPL has mostly positive connections with the regions defining the default mode network, which could be their involvement in higher order of cognitive and attentional tasks (Alahmadi, 2021). Gu and Zhang (2019) reported that patients with MCI showed hyperactive resting-state activity in the SPL, which was consistent with our study. For the DOP group, increased ReHo values of SPL showed significant correlation with OC in the follow-up research. Oury et al. (2013) showed that OC crossed the placenta and the blood-brain barrier to influence fetal development and cognitive function in mice. Puig et al. (2016) indicated that lower serum OC concentrations were associated with brain microstructural changes and worse cognitive performance. Therefore, we speculated that OC as an intermediate of bone metabolism might be the key factor leading to cognitive impairment in osteoporotic patients, and the SPL might be the specific target and functional site.

The PE has important roles in cognitive function, including visuospatial imagery and episodic memory retrieval (Cavanna and Trimble, 2006). Histopathological deposition of amyloid in the PE occurs in the early stage of MCI (Wu et al., 2012), and its resulting damage leads to atrophy of the PE, as seen in early-stage of Alzheimer's disease (AD) (He et al., 2007). The PCC, PE, and parietal lobe were considered to be deeply implicated in the pathophysiology of AD since these regions showed reduced glucose metabolism (Del Sole et al., 2008) and regional cerebral blood flow (rCBF) (Borroni et al., 2006) from an early stage of the disease. In our study, we found the ReHo values in the left PE increased in the DOP group, which suggested that osteoporosis, as a metabolic disease, tended to exacerbate the progression of AD, which could be a predisposing factor for AD progression. A prospective cohort study found that women with faster bone loss were more likely to have cognitive decline (Zhou et al., 2014a), which was consistent with our results.

Interestingly, the multiple abnormal brain regions identified in our study are affiliated with the default mode network (DMN). As a functionally homogeneous system, the DMN involves the posterior cingulate cortex, precuneus, medial prefrontal cortex, and temporal regions, which are the most active at rest and suspended during cognitive activity (McCormick et al., 2014), and are suggested as a major contributor to the normal cognitive functioning (Smucny et al., 2014). Reduction of intrinsic connectivity of the DMN has been observed in several mental disorders, such as AD, autism, schizophrenia, and hepatic encephalopathy (Buckner et al., 2008; Zhang et al., 2012; Zhou et al., 2012; Adriaanse et al., 2014). Our results furtherly confirmed that the default network was the most active network in the resting state. The brain damage caused by osteoporosis firstly affected the DMN, an osteoporosis-exacerbated cognitive impairment based on T2DM.

The preliminary study has some limitations. Firstly, this study had a relatively small sample size. Due to strict inclusion criteria, relatively few patients with DOP were enrolled. Further study with more participants and follow-up on these patients are great importance to evaluate whether the OC could be markers for tracking the very early changes of brain function associated with patients with DOP. Secondly, this study was inadequate to examine the spontaneous activity of DOP by using ReHo without studying structural image data at the same time in resting-state. We should combine multi-modal imaging data to establish these relationships in the future, which would provide more accurate interpretation of the neural mechanisms of cognitive impairment by osteoporosis in T2DM. Moreover, we did not include a completely healthy control group, because the purpose of this study was to observe the effect of osteoporosis on altered spontaneous brain activity in patients with T2DM, rather than the influence of T2DM itself.

## Conclusion

Based on ReHo analysis on rs-fMRI, this study suggested that activities of multiple brain regions were altered in patients with DOP, which indicated that osteoporosis exacerbated cognitive impairment and brain damage. Also, the OC might be considered as a bone marker to track the progression of cognitive impairment.

## Data Availability Statement

The original contributions presented in the study are included in the article/supplementary material, further inquiries can be directed to the corresponding author/s.

## Ethics Statement

The studies involving human participants were reviewed and approved by The Second Affiliated Hospital of Shandong First Medical University. The patients/participants provided their written informed consent to participate in this study.

## Author Contributions

ML: writing—original draft preparation. HY: formal analysis and writing—reviewing and editing. JQ: conceptualization and methodology. XZ: data curation and methodology. QY: visualization and resources. GY: formal analysis, data curation, and software. JuL: visualization and software. JiL: resources, supervision, and project administration. All authors contributed to the article and approved the submitted version.

## Funding

This study was supported by the Medical Health Science and Technology Development Plan Project of Shandong Province (202009041141), the Academic promotion programme of Shandong Frist Medical University (2019QL017), the Medical Health Science and Technology Development Plan Project of Shandong Province (202109010477), and the Science and Technology innovation development project of Tai'an city (2021NS127).

## Conflict of Interest

The authors declare that the research was conducted in the absence of any commercial or financial relationships that could be construed as a potential conflict of interest.

## Publisher's Note

All claims expressed in this article are solely those of the authors and do not necessarily represent those of their affiliated organizations, or those of the publisher, the editors and the reviewers. Any product that may be evaluated in this article, or claim that may be made by its manufacturer, is not guaranteed or endorsed by the publisher.
